# Fostering science–art collaborations: A toolbox of resources

**DOI:** 10.1371/journal.pbio.3001992

**Published:** 2023-02-09

**Authors:** Callie R. Chappell, Louis J. Muglia

**Affiliations:** 1 Department of Biology, Stanford University, Stanford, California, United States of America; 2 Burroughs Wellcome Fund, Research Triangle Park, Durham, North Carolina, United States of America

## Abstract

Scientists and artists are both motivated by creativity and curiosity. Similarly, science and art can be mutually reinforcing, supporting discovery and innovation. This Community Page provides resources for individuals, groups, and institutions to advance science–art collaborations.

## Teaser

Scientists and artists are both motivated by creativity and curiosity, and science and art can be mutually reinforcing, supporting discovery and innovation. This Community Page highlights resources for individuals, groups, and institutions to advance science–art collaborations.

## Introduction

Scientists and artists are both are driven by curiosity and creativity. Curiosity causes both scientists and artists to try and understand and represent the world around them. To answer questions such as “what do we not understand?”, we need creativity. And what we create can help us to better see the world around us. Whether posters, paintings, talks, plays, or papers, both artists and scientists create esthetic products that help us and others to better understand the world [1]. Moreover, both art and science draw on a common toolbox of cognitive approaches [2]. Art is not merely a useful technique for observing and articulating empirical processes, but a creative approach that expands the limits of discovery [3]. By using creative media such as dance, textiles, painting, and sculpture, we can explore scientific questions and communicate our hypotheses and findings in novel ways.

Scientific discovery is an incremental process, but some of the greatest scientific innovations have come from transdisciplinary thinkers that integrate the sciences and the arts. For example, obsidian (*ītztli* in Nahuatl) tools have been used in ancient and modern Mesoamerican art and surgical scalpels [4], classic Japanese illustrated monographs (*Honzou Gaku*) are some of the earliest records of biodiversity [5], and Mae Jemison’s dance background supported her work as an astronaut [6]. Despite these fundamental similarities, art and science are often seen as two cultures [7]. Yet, a dualistic conception of art and science ignores the many scientific advances that arise from synergy between researchers’ artistic and creative endeavors. To address the greatest challenges today, we must inspire and reward work that transcends disciplines.

**Fig 1:**
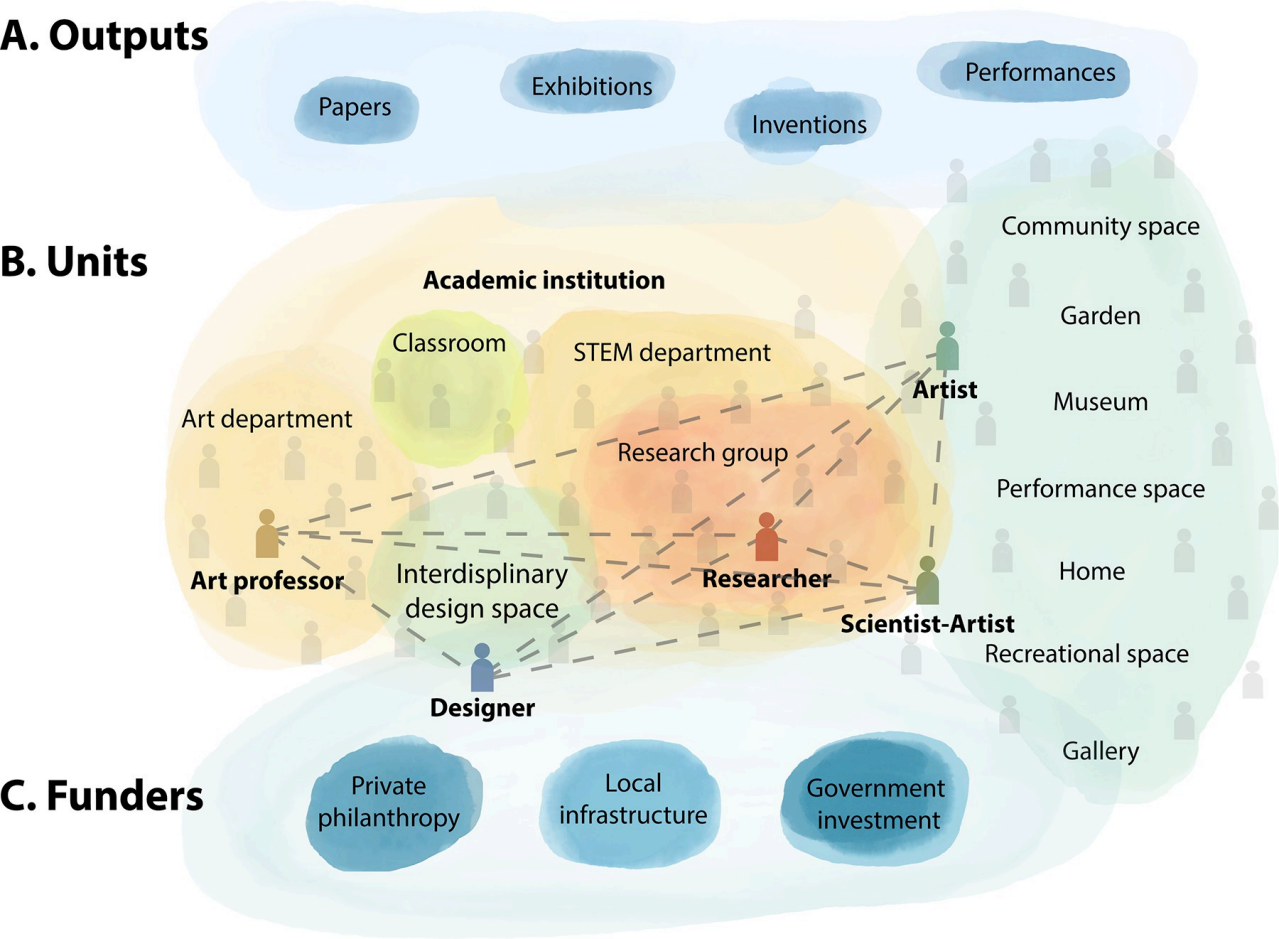
Integrating art and science. (A) Diverse outputs come from science–art collaborations, including papers, exhibitions, inventions, performances, and others. (B) These collaborations can be within academic institutions (such as STEM departments, art departments, in the classroom, or in transdisciplinary spaces such as maker spaces), and outside of academic institutions (such as in community spaces, gardens, museums, performance spaces, recreational spaces, galleries, or even at home). Individuals who move in each of these spaces can be connected, and some can span multiple spaces (in color). (C) Potential funding sources to support this work include private philanthropy, local infrastructure, and government investment.

As both scientists and artists (S1 Text), we believe that expanding practices considered to be science and reframing art as a central dimension of scientific work may yield insightful discoveries and broadly impactful work. In this Community Page, we provide suggestions for how individual researchers can incorporate art into their scientific practices, both artists and scientists can see the commonalities in their approaches, as well as institutional actions academics can take to support art–science collaborations (Fig 1).

## Suggestions for individuals

A straightforward way to integrate art and science is to expand creative practices in research. First, we must see that researchers are artists! In expanding who we consider science practitioners, we can embrace the creativity we all carry. Many science–art collaborations come from a desire to share research findings more broadly, yet graphic illustrations of research are just the tip of the iceberg (Fig 1A). Some researchers create “data sculptures” to summarize their data. Others share their research physically through dance or music (sonification) [8]. Formally integrating artistic media into academic research can yield key insights; for example, Janet Iwasa’s group uses animations to develop visual hypotheses of molecular and cellular processes [9].

Many universities and research institutes already have science–art groups in the form of transdisciplinary journals, science communication groups, science art studio spaces, and professional forums. There are also inter-institutional organizations that support academic researchers who have an interest in integrating art more formally into their research (Table 1). Independent artists and art schools such as the Rhode Island School of Design (RISD) support advanced research and design at the intersection of art and science.

**Table 1:** Resources for integrating art and science.

Opportunity	Example
*Transdisciplinary groups*	
Science–art conferences	**ComSciCon**: Conferences for graduate students focusing on science communication.
Standalone organizations	**Guerilla Science**: Consulting and training for expanding the reach of science.
University centers	**Alan Alda Center for Communicating Science**: University-based center that provides training in science communication.
Individual labs	**Incubator Art Lab:** Art/science laboratory focused on biotechnology and art.
***Funding mechanisms***	
Federal agencies	**Sound Health Initiative:** National Institutes for Health–National Endowments for the Arts collaboration with the John F. Kennedy Center for the Performing Arts and the National Symphony Orchestra.
Universities	**Center for Art, Science, and Technology (CAST):** Massachusetts Institute of Technology and Andrew W. Mellon Foundation center for connecting art, science, and technology.
Foundations	Vanderbilt University’s Institute for Infection, Immunology, and Inflammation **Artist-in-Residence program**, funded by the Burroughs Wellcome Fund.
Multiple funding sources	**BioArt Laboratories** is a physical laboratory and foundation supported by the Netherlands Ministry of Education, Culture, and Science as well as local, private, and state funding sources.
Grants to individuals	**Civic Science Fellows:** Fellowship program supporting individuals working to connect science and society supported by a variety of non-profits and philanthropy organizations.

Researchers can also collaborate with artists, musicians, and educators locally. Artists can work full- or part-time in academic labs, departments, and institutes, learning alongside scientists and producing art inspired by the research they observe. For example, the European Organization for Nuclear Research (CERN) supports artist residencies, commissioned work, and exhibitions through their Arts at CERN program. Similar to research projects, for such collaborations to be productive, both parties must respect the expertise and differences in approaches and perspectives [10]. Scientists must respect the liberty and creativity of the collaborating artist, from the conception of the project to fair compensation for their time, labor, and expertise. To connect with artists, we encourage scientists to explore arts spaces, not only just at museums and galleries, but also at public art openings, community events, gardens, and youth art spaces (Fig 1B). Just with research collaborations, after an initial meeting or email, artists and scientists can develop a project proposal, apply for funding, and create new work together. Approaching conversations with artists with openness, humility, and an enthusiasm to learn will help build trust.

Federal and private sources exist to fund groups that work at the intersection of science and the arts (Fig 1C). In addition to federal funding, universities can work with foundations and non-governmental organizations. Program officers can connect scientists with grant pathways or supplements to support transdisciplinary work. In total, the cost of art–science collaborative efforts, often in the order of hundreds or thousands of dollars, are far less than most scientific research programs. Yet, they can have outsized impacts on the production and dissemination of such work (Table 1).

Similarly, funding exists for individual labs, scientists, and artists to pursue transdisciplinary work. Scientists can write science–art projects into federal grants, as well as applying for supplements that support these broader impacts. Stand-alone federal programs fund collaborations between scientists and artists that are based in specific projects or fund individuals through fellowships (Table 1).

## Suggestions for institutions

Historically, academic departments at universities have trained graduate students and promoted faculty for deep and focused areas of scholarship. While such framing is appropriate, institutional leadership must also prioritize breadth, in addition to depth, of transdisciplinary work between science and arts as equally meritorious. Transdisciplinary collaborations can expand the understanding, public support, and impact of research [11] as well as improve educational outcomes for students [12]. To do so, institutions need to reform the metrics used to assess success in trainees and faculty, as well as invest in venues for transdisciplinary training.

To incentivize transdisciplinary art–science collaboration, academic departments and institutes must revise how they assess and train scientists. For example, universities should offer enhancement experiences for students and faculty at all stages to engage and grow in science–art collaboration, such as seminars, clubs, internships, and awards. Graduate students should be supported in pursuing non-research activities in their PhD, such as art–science exhibitions, community-engaged work, or produce science communication products. For graduate students, these products should be seen as significant contributions to their graduate studies and eligible as dissertation chapters. For faculty, transdisciplinary products should be seen as significant and distinguishing contributions in the tenure evaluation process. Institutional culture should evolve to value and celebrate the “non-traditional” venues in which these products are likely to appear: museums, websites, opinion pieces, theaters, art galleries, and many others (Fig 1A). Building these into existing graduate curricula, additional certification programs, and degrees in science–art integration could be ways to acknowledge the value of these experiences for academics and trainees.

Second, institutions should create new spaces for art–science collaborations and normalize collaborations between artists and scientists happening in both research labs and artist studios (Fig 1B). Although space at universities is at a premium, setting aside catalytic collaborative spaces or workshops for artists and scientists to work together—ultimately leading to a blurring of the boundaries between what is a scientist and what is an artist—should be fostered. This can come through creating artists residencies within science spaces or building transdisciplinary maker/lab/studio spaces, such as the Product Realization Lab at Stanford University (Fig 1C). One way to evaluate success is to what extent participants further engage with STEAM. Such spaces can transform both science and art by centering knowledge from historically excluded groups [13].

## Conclusion

To address the most pressing challenges in STEM, we need to foster a scientific community that centers diverse perspectives and ways of knowing. By amplifying creativity, play, and truly transdisciplinary work, we can create cultural change in the scientific community that is necessary to fuel the discoveries of today and tomorrow.

## Supporting information

S1 Text Biographical information about the co-authors. (DOCX)
