# Supplementary material for: Fostering science–art collaborations: A toolbox of resources
Source: PLoS Biol. 2023 Feb 9;21(2):e3001992. doi: 10.1371/journal.pbio.3001992 (PMC9910691; doi:10.1371/journal.pbio.3001992)
Supplement: S1 Text — (DOCX) [file pbio.3001992.s001.docx]

## S1 Text: Biographical information about the co-authors

## *
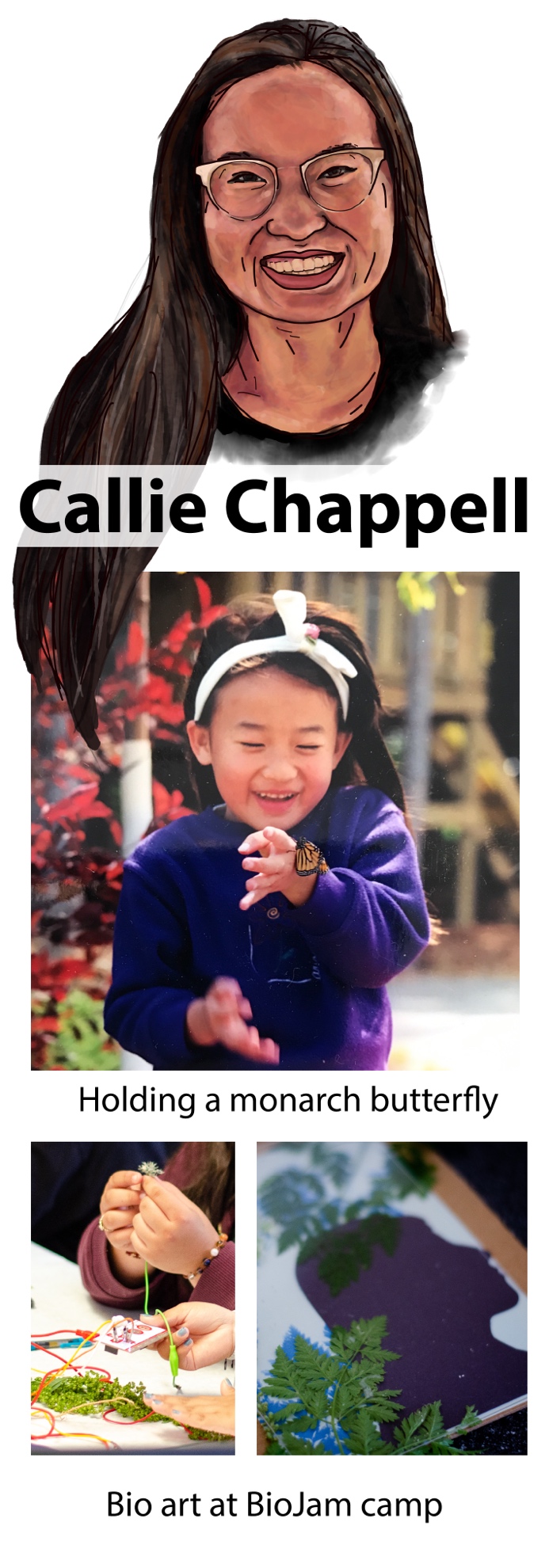
Callie Chappell*

I grew up in the rural Midwest, picking wild apples and running along the shores of Lake Michigan. Living in the countryside, I felt attuned to the seasonal ebb and flow: watching snow melt and leaves burst from buds in spring. The ecology of the land felt aesthetic, which shaped my identity formation as both an artist and scientist. I was fortunate to attend a small, rural charter school that emphasized outdoor education and the intersection of STEM and the arts. That changed in high school, where my science fair poster was “too pretty to be taken seriously” and my undergraduate presentations were underhandedly complimented “as if they were taken from the pages of a magazine.” I was frustrated. I could see that science could be simultaneously beautiful and powerful. Why couldn’t my teachers, professors, and TAs see the same?

As a graduate student, I’ve been fortunate to have an advisor, Tadashi Fukami, that has supported my growth, not just as a scientist, but as a whole person. He encouraged me to integrate my art into the lab’s research, first illustrating figures for a review paper we wrote together, then infographics for lab outreach. Through his mentorship, I felt cared for, not only for my work, but for my personal growth too. This motivated me to get involved in a science communication student organization at my university, hosting science storytelling, video-making, and graphic design workshops. Through this community, I learned that there were a lot of scientists like me: artists not sure how to formally integrate their creativity in the visual arts to support their research. Professionally, this grew into a scientific illustration/graphic design business for scientists, as well as a science communication workshop and consulting practice.

But something felt missing. I saw that much of the conversation around “science communication” assumes that academic researchers are communicating predetermined knowledge to a naïve public. But who is “the public”? I think of the community in which I grew up; many of my peers did not pursue advanced degrees in STEM, but I consider them to be experts. For example, my childhood art teacher, friend and mentor Merrily Bauer taught me how to be a naturalist. She showed me how to look for the catchlight that glances off an eyeball, to capture the subtle, earthy smell of fall leaves, and the iridescence of dew on a fiddlehead fern. Now, as a PhD candidate in Ecology and Evolution, I know that these artistic observational skills are central to being a good ecologist. Even within my own discipline, I need science communication skills to make my research legible to biologists who are not in ecology.

How can scientists learn alongside those who have chosen other life paths (both outside and within academic research) to explore and create together? I have the privilege of learning from Corinne Okada Takara, a community bio artist, Rolando Cruz Perez, a bioengineer and social justice activist, and many others about how to grow spaces where youth and communities historically excluded from academic research can explore, create, and hold communion with other organisms by integrating art and biology. As part of a teen STEAM program called BioJam, we have grown mycelium into quilts with culturally-relevant substrates such as sterilized ramen noodles and nopales cactus, and explored hibiscus and turmeric to dye algae hydrogel-based wires. Rolando, Corinne, and our many collaborators have shown me that art can be a praxis that centers lived experience, culture, and community in the creative, exploratory process of science. I’ve seen more radical innovation prototyping and designing alongside youth than I ever have during my time at Stanford by centering community and culture in both scientific and artistic practice. Together, these experiences have shown me that art not only strengthens science, but is central to it.

#### **
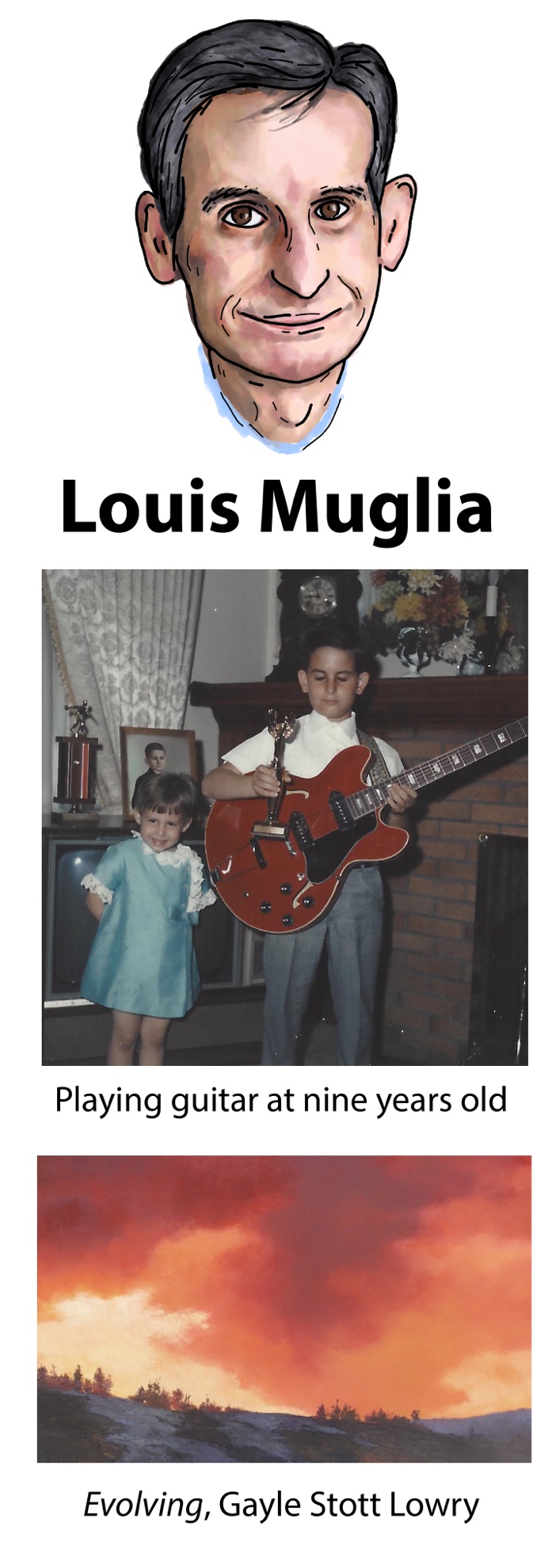
Louis Muglia**

Today, I see art as a critical avenue for creativity and discovery in science, but as a child, I never would have predicted how it would influence my career as a scientist. I grew up in urban Detroit in the 1960’s in a working class Italian family. I was the first to go to college. While neither artists nor scientists, my parents prioritized the importance of education for my siblings and me and encouraged me to start playing the guitar. I enjoyed music theory, the “mathematical” relationship between notes that generates chords of distinct character. During high school, I played and taught jazz guitar while balancing my interests in academics. At the time, I felt I had to code-switch between my “cool” music friends and my passion for learning, especially math and science. As an undergraduate, I played guitar in a new wave/punk band, The Zero’s, and started pursuing biomedical research. Studying to The Clash, Patti Smith, and The Ramones heightened my awareness of how sound influences mind, body and mood–and sometimes revealed solutions to classroom assignments! The ways sound and statement in the music connected with how my brain processed information to generate joy or sadness intrigued me and led me to study neuroscience.

I have spent most of my 40-year academic career as a physician-scientist, for the last 30 years focusing on understanding the pathogenesis of adverse pregnancy outcomes, their consequences particularly for neurodevelopment, and how they are shaped by biological and social factors. I have learned that we cannot move the needle on critical health issues without engaging, partnering and learning from communities. To do so, I have come to appreciate the power of storytelling, images, and the arts in fostering understanding and shared humanity. Because of my passion for music, poetry and visual arts, I see the potential of integrating science and the arts to create healthcare systems that better serve excluded populations, create spaces for reciprocal and equitable learning, and inspire the next generation of creative discoverers.

To accomplish this goal, I have transitioned in my career from running a basic science/translational laboratory to being President and CEO of a science philanthropy organization. In this role, I hope to address the determinant of our greatest health issues by supporting talented individuals driving an agenda for positive change. To foster diversity, community engagement, and the beauty and promise of science, integrating science and the arts has untapped potential to benefit society. I hope to help develop sustainable support mechanisms and environments for the scientist-artists and artist-scientists that are passionate about working at this interface. During my tenure as CEO, we have worked with partner organizations to support several science-artists including Anand Varma’s biological photography, Alvaro Laiz’s photo documentary of human migration, and Peter Sellars’ multi-disciplinary film about health, illness and physical fragility in the context of COVID-19. I hope to see a new career path for artists and scientists that use multi-media representation - images, sounds, and words - to enhance and clarify scientific information. This scholarship can be supported both in academia and publicly to convey data and demonstrate the human toll and resilience associated with emerging diseases, particularly those associated with climate change and human health.

Outside of my professional work, I am personally enthusiastic about supporting local and emerging artists to create inspirational pieces that marry art and science. For example, Gayle Stott Lowry conveys a deep and urgent sense of humanity’s impact on climate and the environment. I commissioned a painting of hers, entitled “Witnessing”: the trees, representing humankind, overlooking a hardened landscape on the background of a fiery sky provide a motivating image around potential future consequences of climate change. Even though I am not a visual artist, I support those who integrate the sciences and arts in my professional and personal life.
